# Social tagging in the life sciences: characterizing a new metadata resource for bioinformatics

**DOI:** 10.1186/1471-2105-10-313

**Published:** 2009-09-25

**Authors:** Benjamin M Good, Joseph T Tennis, Mark D Wilkinson

**Affiliations:** 1 Heart + Lung Institute at St. Paul's Hospital, University of British Columbia, Vancouver, Canada; 2 Information School, University of Washington, Seattle, USA

## Abstract

**Background:**

Academic social tagging systems, such as Connotea and CiteULike, provide researchers with a means to organize personal collections of online references with keywords (tags) and to share these collections with others. One of the side-effects of the operation of these systems is the generation of large, publicly accessible metadata repositories describing the resources in the collections. In light of the well-known expansion of information in the life sciences and the need for metadata to enhance its value, these repositories present a potentially valuable new resource for application developers. Here we characterize the current contents of two scientifically relevant metadata repositories created through social tagging. This investigation helps to establish how such socially constructed metadata might be used as it stands currently and to suggest ways that new social tagging systems might be designed that would yield better aggregate products.

**Results:**

We assessed the metadata that users of CiteULike and Connotea associated with citations in PubMed with the following metrics: coverage of the document space, density of metadata (tags) per document, rates of inter-annotator agreement, and rates of agreement with MeSH indexing. CiteULike and Connotea were very similar on all of the measurements. In comparison to PubMed, document coverage and per-document metadata density were much lower for the social tagging systems. Inter-annotator agreement within the social tagging systems and the agreement between the aggregated social tagging metadata and MeSH indexing was low though the latter could be increased through voting.

**Conclusion:**

The most promising uses of metadata from current academic social tagging repositories will be those that find ways to utilize the novel relationships between users, tags, and documents exposed through these systems. For more traditional kinds of indexing-based applications (such as keyword-based search) to benefit substantially from socially generated metadata in the life sciences, more documents need to be tagged and more tags are needed for each document. These issues may be addressed both by finding ways to attract more users to current systems and by creating new user interfaces that encourage more collectively useful individual tagging behaviour.

## Background

As the volume of data in various forms continues to expand in the life sciences and elsewhere, it is increasingly important to find mechanisms to generate high quality metadata rapidly and inexpensively. This indexing information - the subjects linked to documents, the functions annotated for proteins, the characteristics identified in images, *etc*. - is what makes it possible to build the software required to provide researchers with the ability to find, integrate, and interact effectively with distributed scientific information.

Current practices for generating metadata within the life sciences, though varying across initiatives and often augmented by automated techniques, generally follow a process closely resembling that long employed by practitioners in the library and information sciences [1,2]. First, semantic structures, such as thesauri and ontologies, are created by teams of life scientists working in cooperation with experts in knowledge representation or by individuals with expertise in both areas. Next, annotation pipelines are created whereby professional annotators utilize the relevant semantic structures to describe the entities in their domain. Those annotations are then stored in a database that is made available to the public via websites and sometimes Web services. As time goes on, the semantic structures and the annotations are updated based on feedback from the community and from the annotators themselves.

This process yields useful results, but it is intensive in its utilization of an inherently limited supply of professional annotators. As the technology to produce new information and the capacity to derive new knowledge from that information increases, so too must the capacity for metadata provision. Technologies that support this process by partially automating it, such as workflows for genome annotation [3] and natural language indexing systems [4-6], provide important help in this regard, but manual review of automated predictions remains critical in most domains [7,8]. There is clearly a need for an increase in the number of human annotators to go along with the increase in the amount of data.

**Figure 1:**
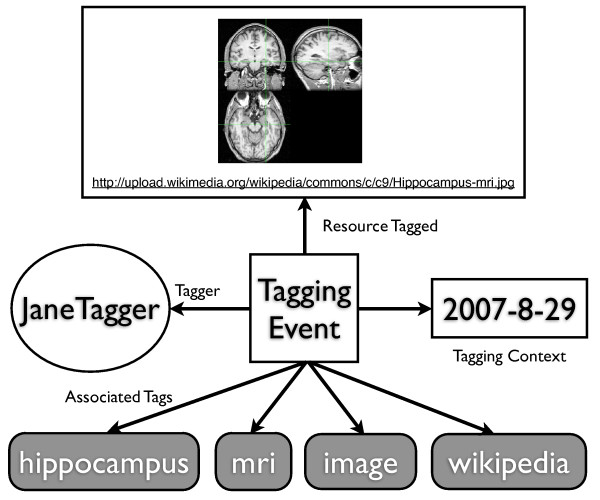
**Data captured in tagging events (posts)**. Tagging events capture information about: the resource tagged, the tagger, the time the event took place, and the tags associated with the resource by the tagger.

Serendipitously, social Web applications such as Connotea [9] and CiteULike [10] are now enabling the emergence of an expanding pool of human annotators - albeit annotators acting to fulfil widely varying purposes and in possession of a broad range of expertise. Connotea and CiteULike are examples of what are known as 'social tagging systems'. Such systems let their users organize personal resource collections with tags (keywords). The kinds of resources contained within them are essentially unlimited, with popular examples including Web bookmarks [11], images [12], and even personal goals [13]. These resource collections are made available to the social network of their creators and often to the general public. The tags used to organize the collections are created by the owner of the collection (the tagger) and can serve a variety of purposes [14]. The act of adding a resource to a social tagging collection is referred to as a 'tagging event' or simply as a 'post' (as in "to post a bookmark"). Figure 1 illustrates the information captured in a record of a typical tagging event in which JaneTagger tags an image retrieved from Wikipedia with the tags 'hippocampus', 'image', 'mri', and 'wikipedia'. Academic social tagging systems, such as Connotea, Bibsonomy and CiteULike, extend this basic functionality with the ability to identify and store bibliographic information associated with scientific articles [9,10,15,16].

The tagline of Connotea - "Organize, Share, Discover" - illustrates the purposes that social tagging systems are intended to enable for their users. Tags can be used to *organize *personal collections in a flexible, location independent manner. The online nature of these services allows users to easily *share *these collections with others - either within their circle of associates or with the general public. Through the public sharing of these annotated references, it is possible for users to *discover *other users with similar interests and references they may not otherwise have come across.

This basic functionality has already attracted tens of thousands of users to these systems.

The expanding numbers of users and the concomitant increase in the volume of the metadata they produce suggests the possibility of new applications that build on the socially generated metadata to achieve purposes different from the personal ones listed above. For example, [17] showed that the relevance of Web search results achieved with both search engines and hand-curated directories could be improved by integrating results produced by social tagging services. In fact, the "social search engines" suggested by this result are already starting to appear (for an example, see worio.com [18]).

As we consider the creation of new applications like these within the life sciences, it is important to begin with an understanding of the nature of the metadata that they will be built upon. This study is thus intended to provide a thorough characterization of the current products of social tagging services in biomedical, academic contexts. This is achieved through an empirical assessment of the tags used to describe citations in PubMed by users of Connotea and CiteULike. Selecting PubMed citations as the resource-focus for this investigation makes it possible to compare socially generated metadata, produced initially to support disparate personal needs, directly with professionally generated metadata produced for the express purpose of enabling applications that serve the whole community. Where commonalities are noted, similar kinds of community-level uses can be imagined for the socially generated metadata; where differences occur, opportunities are raised to envision new applications.

## Results

### Resource Coverage

**Figure 2:**
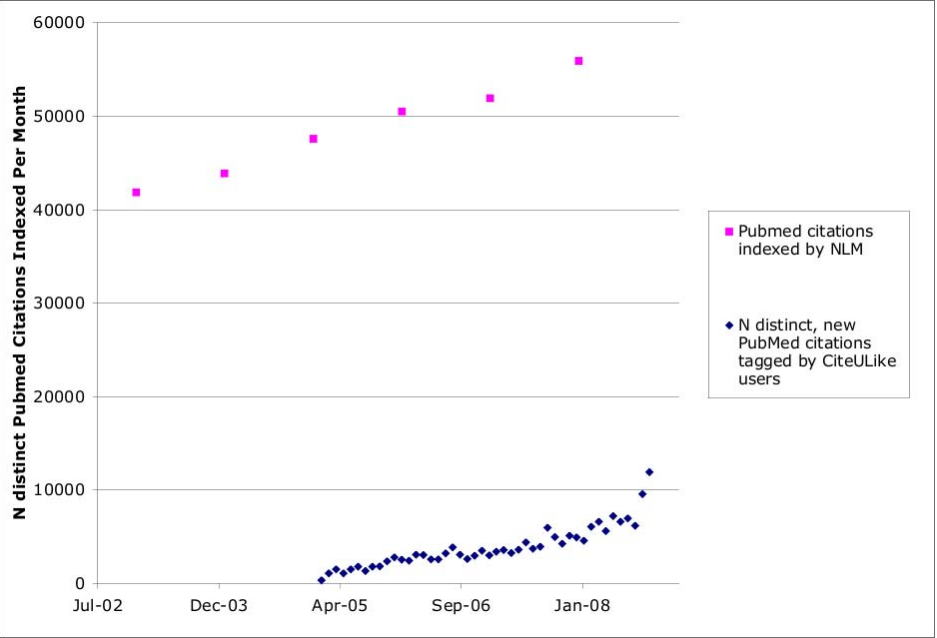
**The number of distinct new biomedical citations indexed per month by CiteULike and by MEDLINE**. The figure illustrates the increasing rates of growth, per month, of new citations with PubMed identifiers to be indexed by MEDLINE (upper points in pink) and tagged by users of CiteULike (lower points in blue). The numbers for MEDLINE were estimated by taking the reported yearly totals and dividing by 12. The numbers for CiteULike were measured directly from a database export.

In the life sciences, the total number of items described by social tagging systems is currently tiny in comparison to the number of resources described by institutions. To illustrate, the MEDLINE bibliographic database contains over 16 million citations [19] while, as of November 9, 2008, CiteULike, the largest of the academic social tagging services, contained references to only about 203,314 of these documents. Figure 2 plots estimates of the numbers of new citations (with distinct PubMed identifiers) added to both PubMed and CiteULike per month over the past several years. The chart provides a visualization of both the current difference in scale and an indication of the rates of growth of both systems. It shows that both systems are indexing more items every month, that CiteULike appears to be growing slightly faster then PubMed, and that CiteULike is approaching 10,000 unique PubMed citations added per month with MEDLINE approaching 60,000.

This data suggests that, despite the very large numbers of registered users of academically-focused social tagging services - on November 10, 2008, Connotea reported more than 60,000 (Ian Mulvaney, personal communication) - the actual volume of metadata generated by these systems remains quite low. While the sheer numbers of users of these systems renders it possible that this volume could increase dramatically, that possibility remains to be shown.

### Density

Density refers simply to the number of metadata terms associated with each resource described. Though providing no direct evidence of the quality of the metadata, it helps to form a descriptive picture of the contents of metadata repositories that can serve as a starting point for exploratory comparative analyses. To gain insight into the relative density of tags used to describe citations in academic social tagging services, we conducted a comparison of the number of distinct tags per PubMed citation for a set of 19,118 unique citations described by both Connotea and CiteULike. This set represents the complete intersection of 203,314 PubMed citations identified in the CiteULike data and 106,828 PubMed citations found in Connotea.

Table 1 provides an assessment of the density of distinct tags used to describe these citations by individual users and by the aggregate of all users of the system. These numbers are contrasted with the average numbers of MeSH subject descriptors (both major and minor subject headings were included) used to index the same set of documents. Only the MeSH descriptors are reported (ignoring large amounts of additional subject-related metadata such as descriptor modifiers, supplementary concept records, and links to other databases such as NCBI Gene [20]).

**Table 1:** Tag density in Connotea, CiteULike and MEDLINE on PubMed citations

**System**	**N sampled**	**mean**	**median**	**min**	**max**	**stand. dev.**	**coefficient of variation**
Connotea per tagging	28236	3.02	2	1	115	3.74	1.24

CiteULike per tagging	45525	2.51	2	0	44	2.16	0.86

Connotea aggregate	19118	4.15	3	1	119	5.14	1.24

CiteULike aggregate	19118	5.1	4	0	74	5.29	1.04

MEDLINE	19118	11.58	11	0	42	5.3	0.46

'N sampled' refers to the number of tagged citations considered. For example, the first row shows the statistics for the number of tags associated with distinct posts to the Connotea service. In contrast, the 'Connotea aggregate' row merges all the posts for each citation into one. In the aggregate cases, the numbers of tags reported refer to the number of *distinct *tags -- repeats are not counted.

In terms of tags per post, the users of CiteULike and Connotea were very similar. As Table 1 indicates, the mean number of tags added per biomedical document by individual users was 3.02 for Connotea and 2.51 for CiteULike, with a median of 2 tags/document for both systems. These figures are consistent with tagging behaviour observed throughout both systems and with earlier findings on a smaller sample from CiteULike which indicated that users typically employ 1-3 tags per resource [21,22]. On independent samples of 500,000 posts (tagging events) for both CiteULike and for Connotea, including posts on a wide variety of subjects, the medians for both systems were again 2 tags/document and the means were 2.39 tags/document for CiteULike and 3.36 for Connotea. The difference in means is driven, to some extent, by the fact that CiteULike allows users to post bookmarks to their collections without adding any tags while Connotea requires a minimum of one tag per post. Other factors that could influence observed differences are that the user populations for the two systems are not identical nor are the interfaces used to author the tags. In fact, given the many potential differences, the observed similarity in tagging behaviour across the two systems is striking.

As more individuals tag any given document, more distinct tags are assigned to it. After aggregating all of the tags added to each of the citations in the sample by all of the different users to tag each citation, the mean number of distinct tags/citation for Connotea was 4.15 and the mean number for CiteULike was 5.10. This difference is a reflection of the larger number of posts describing the citations under consideration by the CiteULike service. In total, 45,525 CiteULike tagging events produced tags for the citations under consideration while data from just 28,236 Connotea tagging events were considered.

**Figure 3:**
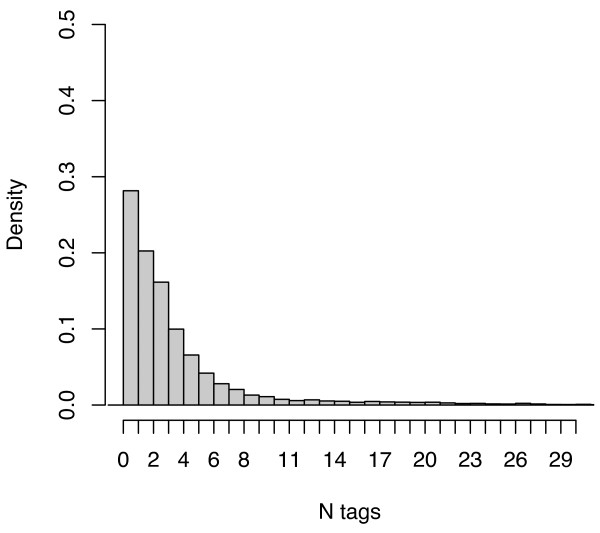
**The number of distinct tags assigned per PubMed citation by the aggregate of Connotea users**. The figure provides a probability density histogram of the number of distinct tags per PubMed citation within Connotea. For each citation, the non-redundant set of tags assigned to that citation by all of the users to post it to their collections is counted. The peak of the distribution (just under a density of 0.3) is at one tag per citation (all Connotea posts have at least one tag) and it drops off smoothly as the number of tags per citation increases down to negligible number beyond about 11 tags per document.

**Figure 4:**
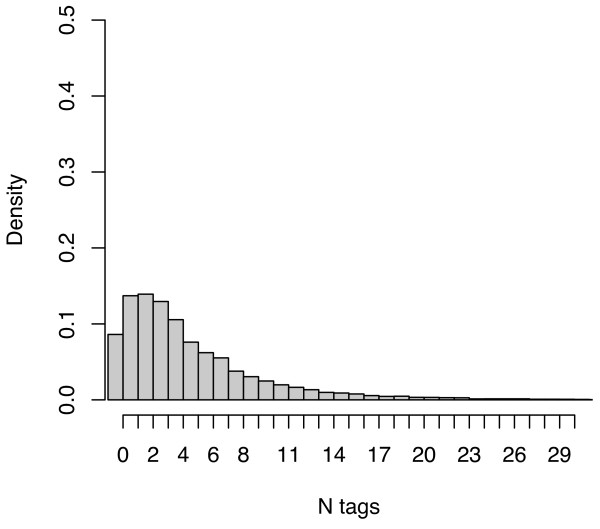
**The number of distinct tags assigned per PubMed citation by the aggregate of CiteULike users**. The figure provides a probability density histogram of the number of distinct tags per PubMed citation within CiteULike. For each citation, the non-redundant set of tags assigned to that citation by all of the users to post it to their collections is counted. The peak of the distribution (just under a density of 0.15) is at two tags per citation with nearly equivalent densities at one and three tags per citation. Beyond three tags, the distribution drops off smoothly with densities becoming negligible beyond about 20 tags per document.

**Figure 5:**
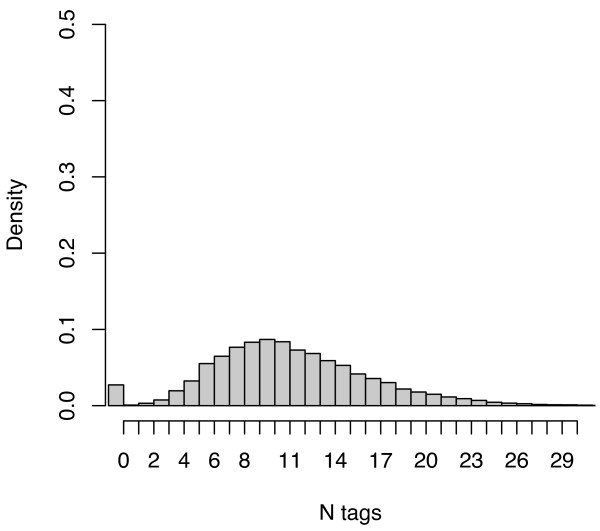
**The number of MeSH subject descriptors assigned per PubMed citation by MEDLINE**. The figure provides a probability density histogram of the number of MeSH subject descriptors assigned per PubMed citation. The peak (just under a density of 0.1) is at 10 descriptors with a near-normal (skewed slightly towards 0) distribution falling away from that maximum to negligible numbers of descriptors at 1 tag per document and about 26 tags document. The one exception to the smooth distribution is an outlier group at zero descriptors per citation with a density of approximately 0.025.

Overall, the subject descriptors from MEDLINE exhibited a much higher density, at a mean of 11.58 and median of 11 descriptors per citation, than the social tagging systems as well as a lower coefficient of variation across citations. Figures 3, 4 and 5 plot the distribution of tag densities for Connotea, CiteULike, and MEDLINE respectively. From these figures we can see that even after aggregating the tags produced by all of the users, most of the citations in the social tagging systems are described with only a few distinct tags. Note that the first bar in the charts shows the fraction of citations with zero tags (none for Connotea).

**Figure 6:**
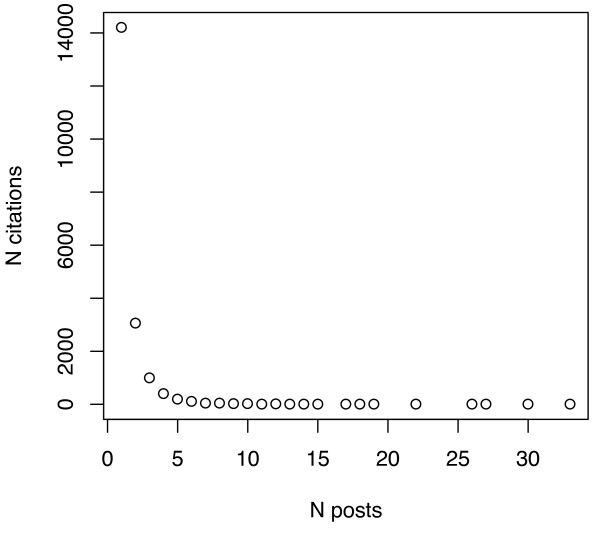
**Relationship between number of PubMed citations and number of Connotea posts per citation**. The X coordinates of each point on the plot correspond to the number of different people to post a particular citation. The Y coordinates are counts of the number citations that occur in each of the bins provided by the number of posts. The point at the upper left of the chart shows that more than 14,000 citations (of a sample of 19,118 unique citations) were only posted by one user. This number decreases exponentially as the number of users to post each citation increases.

**Figure 7:**
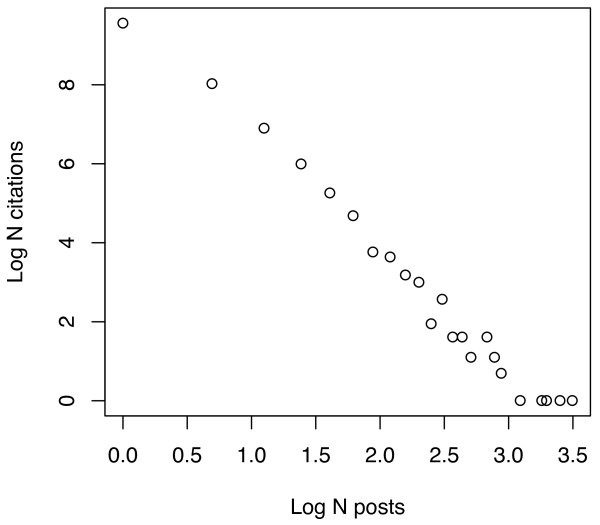
**Relationship between number of PubMed citations and number of Connotea posts per citation plotted on a log-log scale**. The X coordinates of each point on the plot correspond to the Log of the number of different people to post a particular citation. The Y coordinates are the Log of the counts of the number citations that occur in each of the bins provided by the number of posts. The near linearity chart illustrates the exponential relationship between the number of citations and the number of Connotea users to post a citation -- a few citations are posted by many people but most are only posted by a few.

**Figure 8:**
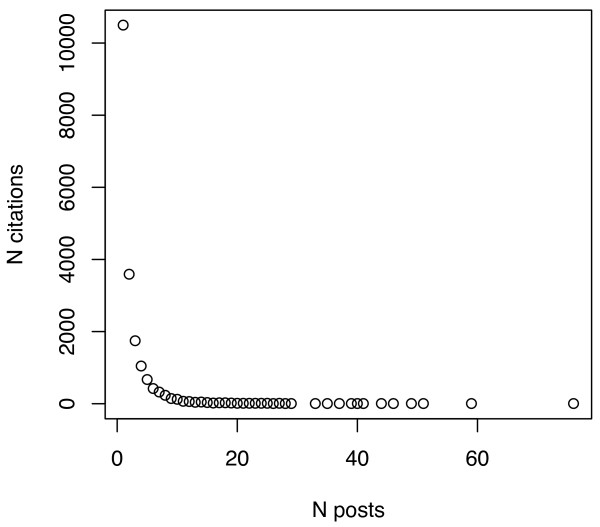
**Relationship between number of PubMed citations and number of CiteULike posts per citation**. The X coordinates of each point on the plot correspond to the number of different people to post a particular citation. The Y coordinates are counts of the number citations that occur in each of the bins provided by the number of posts. The point at the upper left of the chart shows that more than 10,000 citations (of a sample of 19,118 unique citations) were only posted by one user. This number decreases exponentially as the number of users to post each citation increases.

**Figure 9:**
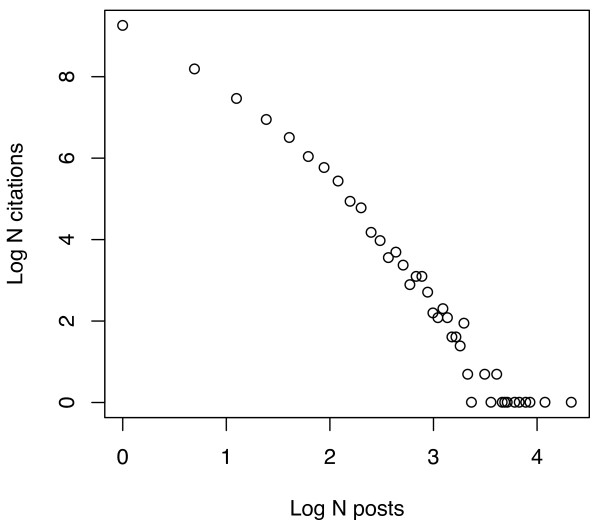
**Relationship between number of PubMed citations and number of CiteULike posts per citation plotted on a log-log scale**. The X coordinates of each point on the plot correspond to the Log of the number of different people to post a particular citation. The Y coordinates are the Log of the counts of the number citations that occur in each of the bins provided by the number of posts. The near linearity chart illustrates the exponential relationship between the number of citations and the number of CiteULike users to post a citation -- a few citations are posted by many people but most are only posted by a few.

One of the reasons for the low numbers of tags/citation, even in the aggregate sets, is that most citations are tagged by just one person, though a few are tagged by very many. To illustrate, Figures 6, 7, 8 and 9 plot the number of citations versus the number of users to post each citation in the Connotea-CiteULike-MEDLINE intersection. Figures 6 and 7 show the data from Connotea on both a linear (Figure 6) and logarithmic scale (Figure 7) and Figures 8 and 9 show the equivalent data from CiteULike. The plots clearly indicate exponential relationships between the number of resources and the number of times each resource is tagged that are consistent with previous studies of the structure of collaborative tagging systems [14].

**Figure 10:**
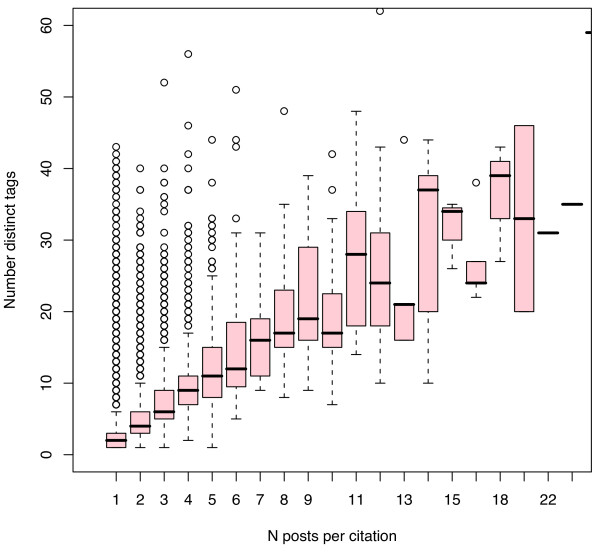
**Increase in tag density per PubMed citation with increase in number of Connotea posts per citation**. Each vertical box and whisker plot describes the distribution of the number of distinct Connotea tags associated with PubMed citations tagged by the number of people indicated on the X axis. For example, the first plot, at X = 1, describes the density of tags per citation assigned by just one person while the second plot, at X = 2, describes the density of distinct tags per citation assigned by the aggregated tags of 2 people and so forth. The median of the distribution is indicated by the horizontal line, the upper and lower boundaries of the box indicate the medians of the first and third quartiles (such that 50% of the data lies within those boundaries), the whiskers extend either to the extremes of the observations or a maximum of 1.5 times the interquartile range, and circles indicate outliers.

**Figure 11:**
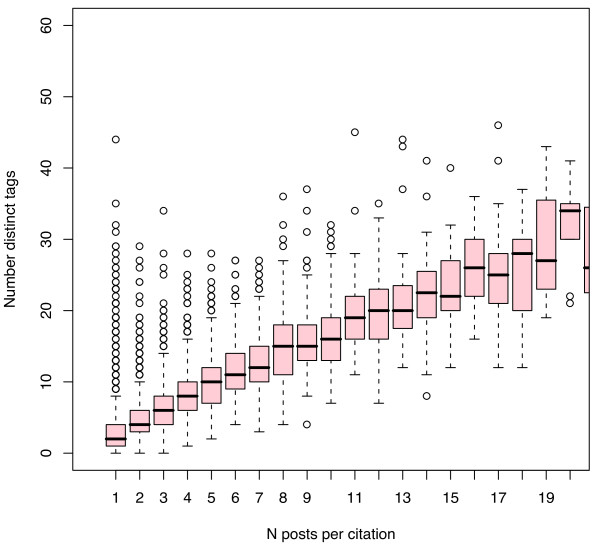
**Increase in tag density per PubMed citation with increase in number of CiteULike posts per citation**. Each vertical box and whisker plot describes the distribution of the number of distinct CiteULike tags associated with PubMed citations tagged by the number of people indicated on the X axis. For example, the first plot, at X = 1, describes the density of tags per citation assigned by just one person while the second plot, at X = 2, describes the density of distinct tags per citation assigned by the aggregated tags of 2 people and so forth. The median of the distribution is indicated by the horizontal line, the upper and lower boundaries of the box indicate the medians of the first and third quartiles (such that 50% of the data lies within those boundaries), the whiskers extend either to the extremes of the observations or a maximum of 1.5 times the interquartile range, and circles indicate outliers.

Current levels of tag density are indicative, but the rates of change provide more important insights regarding the potential of these young systems. Figures 10 and 11 plot the increase in distinct tags/citation as more Connotea (Figure 10) and CiteULike (Figure 11) users tag PubMed citations. These figures suggest that in order to reach the same density of distinct tags per resource as MeSH descriptors per resource produced by MEDLINE (median 11), roughly 5 to 7 social taggers would need to tag each citation. Since, at any given time it appears that the vast majority of citations will be described by just one person, as indicated in Figures 6, 7, 8 and 9, the data suggests that the density of distinct socially generated tags used to describe academic documents in the life sciences will remain substantially lower than the density of institutionally created subject descriptors. This prediction is, of course, dependent on current parameters used for the implementations of academic social tagging systems. As interfaces for adding tags change, the density of tags per post as well as the level of agreement between the different taggers regarding tag assignments may change.

### Inter-annotator agreement

Measures of inter-annotator agreement quantify the level of consensus regarding annotations created by multiple annotators. Where consensus is assumed to indicate correctness, it is used as measure of quality. The higher the agreement between multiple annotators, the higher the perceived confidence in the annotations.

In a social tagging scenario, agreement regarding the tags assigned to particular resources can serve as a rough estimate of the quality of those tags from the perspective of their likelihood to be useful to people other than their authors. When the same tag is used by multiple people to describe the same thing, it is more likely to directly pertain to the important characteristics of the item tagged (*e.g*. 'VEGF' or 'solid organ transplantation') than to be of a personal or erroneous nature (*e.g*. 'BIOLS_101', 'todo', or '**'). Rates of inter-annotator agreement can thus be used as an approximation of the quality of tag assignments from the community perspective. Note that, as [23] discusses, there may be interesting, community-level uses for other kinds of tags, such as those bearing emotional content. For example, tags like 'cool' or 'important' may be useful in the formation of recommendation systems as implicit positive ratings of content. However, the focus of the present study is on the detection and assessment of tags from the perspective of subject-based indexing. Note also that the small numbers of tags per document in the systems under consideration here bring into question the relationship between consensus and quality.

To gauge levels of inter-annotator agreement, we calculate the average level of positive specific agreement (PSA) regarding tag assignments between different users [24]. PSA is a measure of the degree of overlap between two sets - for example, the sets of tags used to describe the same document by two different people. It ranges from 0, indicating no overlap, to 1, indicating complete overlap. (See the Methods section for a complete description.) For this study, we measured PSA for tag assignments at five different levels of granularity: string, standardized string, UMLS concept, UMLS semantic type, and UMLS semantic group. At the first level, PSA is a measurement of the average likelihood that two people will tag a document with exactly the same string of characters. At the next level, we measure the likelihood that two people will tag the same resource with strings of characters that, after syntactic standardization (described in the Methods section), are again exactly the same. Moving up to the level of concepts, we assess the chances that pairs of people will use tags that a) can be mapped automatically to concept definitions in the UMLS and b) map to the same concepts. (Note that not all of the tags in the sample were successfully mapped to UMLS concepts; only tagging events where at least one UMLS concept was identified were considered for the concept, type, and group level comparisons.) At the level of semantic types, we are measuring the degree to which pairs of taggers are using the same basic kinds of concepts where these kinds are each one of the 135 semantic types that compose the nodes of the UMLS semantic network [25,26]. At the uppermost level, we again measure the agreement regarding the kinds of tags used, but here, these kinds are drawn from just 15 top-level semantic groups designed to provide a coarse-grained division of all of the concepts in the UMLS [27]. Table 2 provides examples from each of these levels.

**Table 2:** Examples of different levels of granularity

**Level**	**Example**
String	'Adolescent-Psychology'

Standardized String	'adolescent psychology'

UMLS Concept	CUI 0001584: 'Adolescent Psychology'

UMLS Semantic Type	SUI T090: 'Biomedical Occupation or Discipline'

UMLS Semantic Group	OCCU: 'Occupations'

The reason for including multiple levels of granularity in the measures of agreement is to provide a thorough comparison of the *meanings *of the tags. Since the tags are created dynamically by users entering simple strings of text, we expect large amounts of variation in the representations of the same concepts due to the presence of synonyms, spelling errors, differences in punctuation, differences in plural versus singular forms, *etc*. The mapping to UMLS concepts should help to reduce the possibility of such non-semantic variations masking real conceptual agreements. Furthermore, by including analyses at the levels of semantic types and semantic groups, we can detect potential conceptual similarities that exact concept matching would not reveal. (While the present study is focused on measures of agreement, in future work this data could be used to pose questions regarding the semantic content of different collections of tags - for example, it would be possible to see if a particular semantic group like 'concepts and ideas' was over-represented in one group versus another.)

Table 3 captures the average levels of PSA observed for CiteULike and Connotea users on taggings of PubMed citations. It shows that average PSA among CiteULike taggers ranged from a minimum of 0.11 at the level of the String to a maximum of 0.52 at the level of the Semantic Group with Connotea users following a very similar trajectory. Table 3 also again illustrates the low numbers of tags per post in the social tagging data and the even lower number of UMLS Concepts that could be confidently associated with the tags. The majority of the posts from both social tagging services contained no tags that could be linked to UMLS concepts. For those posts for which at least one Concept was identified, means of just 1.39 UMLS Concepts per post were identified in CiteULike and 1.86 in Connotea.

**Table 3:** Positive Specific Agreement among pairs of social taggers on PubMed citations

	**CiteULike**			**Connotea**		
	
	**Mean PSA**	**N pairs measured**	**Mean terms per post**	**Mean PSA**	**N pairs measured**	**Mean terms per post**
**String**	0.11	19782	2.49	0.14	13156	3.06

**Standardized String**	0.13	19782	2.49	0.16	13156	3.06

**Concepts**	0.39	9128	1.39	0.31	4022	1.86

**Types**	0.43	9128	1.36	0.38	4022	1.72

**Groups**	0.52	9128	1.29	0.45	4022	1.56

One interpretation of the low levels of agreement is that some users are providing incorrect descriptions of the citations. Another interpretation is that there are many concepts that could be used to correctly describe each citation and that different users identified different, yet equally valid, concepts. Given the complex nature of scientific documents and the low number of concepts identified per post, the second interpretation is tempting. Perhaps the different social taggers provide different, but generally valid views on the concepts of importance for the description of these documents. If that is the case, then, for items tagged by many different people, the aggregation of the many different views would provide a conceptually multi-faceted, generally correct description of each tagged item. Furthermore, in cases where conceptual overlap does occur, strength is added to the assertion of the correctness of the overlapping concepts.

To test both of these assumptions, some way of measuring 'correctness' regarding tag assignments is required. In the next several sections, we offer comparisons between socially generated tags and the MeSH subject descriptors used to describe the same documents. Where MeSH annotation is considered to be correct, the provided levels of agreement can be taken as estimates of tag quality; however, as will be shown in the anecdote that concludes the results section and addressed further in the Discussion section, MeSH indexing is not and could not be exhaustive in identifying relevant concepts nor perfect in assigning descriptors within the limits of its controlled vocabulary. There are likely many tags that are relevant to the subject matter of the documents they are linked to yet do not appear in the MeSH indexing; agreement with MeSH indexing can not be taken as an absolute measure of quality - it is merely one of many potential indicators.

### Agreement with MeSH indexing

As both another approach to quality assessment and a means to precisely gauge the relationship between socially generated and professionally generated metadata in this context, we compared the tags added to PubMed citations to the MeSH descriptors added to the same documents. For these comparisons, we again used PSA, but in addition, we report the precision and the recall of the tags generated by the social tagging services with respect to the MeSH descriptors. (For readers familiar with machine learning or information retrieval studies, in cases such as this where one set is considered to contain true positives while the other is considered to contain predicted positives, PSA is equivalent to the F measure - the harmonic mean of precision and recall.)

For each of the PubMed citations in both CiteULike and Connotea, we assessed a) the PSA, b) the precision, and c) the recall for tag assignments in comparison to MeSH terms at the same five semantic levels used for measuring inter-annotator agreement. For each PubMed citation investigated, we compared the aggregate of all the distinct tags added by users of the social tagging service in question to describe that citation with its MeSH descriptors. Table 4 provides the results for both systems at each level. It shows how the degree of agreement with MeSH indexing increases as the semantic granularity at which the comparisons are made widens. As should be expected based on the much lower numbers of UMLS Concepts associated with the social tagging events, the recall is much lower than precision at each level.

**Table 4:** Average agreement between social tagging aggregates and MeSH indexing.

	**CiteULike verse MEDLINE**				**Connotea verse MEDLINE**			
	
	**N Citations**	**Mean precision**	**Mean recall**	**Mean PSA**	**N Citations**	**Mean precision**	**Mean recall**	**Mean PSA**
**String**	19059	0	0	0	19118	0.03	0.02	0.02

**Normalized String**	19059	0.09	0.03	0.04	19118	0.10	0.04	0.05

**Concepts**	8933	0.20	0.02	0.03	9290	0.30	0.04	0.07

**Types**	8933	0.56	0.07	0.12	9290	0.59	0.10	0.16

**Groups**	8933	0.81	0.18	0.29	9290	0.81	0.22	0.32

Focusing specifically on precision, we see that approximately 80% of the concepts that could be identified in both social tagging data sets fell into UMLS Semantic Groups represented by UMLS Concepts linked to the MeSH descriptors for the same resources. At the level of the Semantic Types, 59% and 56% of the kinds of concepts identified in the Connotea and CiteULike tags respectively, were found in the MeSH annotations. Finally, at the level of UMLS Concepts, just 30% and 20% of the concepts identified in the Connotea and CiteULike tags matched Concepts from the MeSH annotations.

### Improving agreement with MeSH through voting

The data in Table 4 represents the conceptual relationships between MeSH indexing and the complete, unfiltered collection of tagging events in CiteULike and Connotea. In certain applications, it may be beneficial to identify tag assignments likely to bear a greater similarity to a standard like this - for example, to filter out spam or to rank search result lists. One method for generating such information in situations where many different opinions are present is voting. Assuming that there is a greater tendency for tag assignments to agree with the standard than to disagree - where multiple tag assignments for a particular document are present - then the more times a tag is used to describe a particular document the more likely that tag is to match the standard.

**Figure 12:**
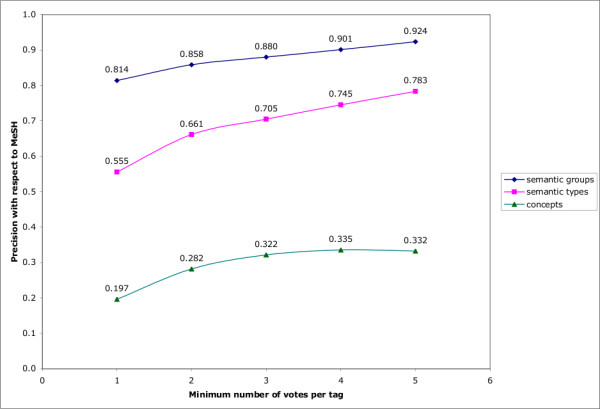
**Precision increase and coverage decrease with voting in CiteULike**. The X axis indicates the minimum number of times a given UMLS Concept (in green), Semantic Type (in pink), or Semantic Group (in dark blue), would need to be associated with a PubMed citation (through the assignment of a tag by a CiteULike user that could be linked to the Concept) to be considered. The Y axis plots the precision with which these different voted aggregates predict the corresponding MeSH annotations.

**Figure 13:**
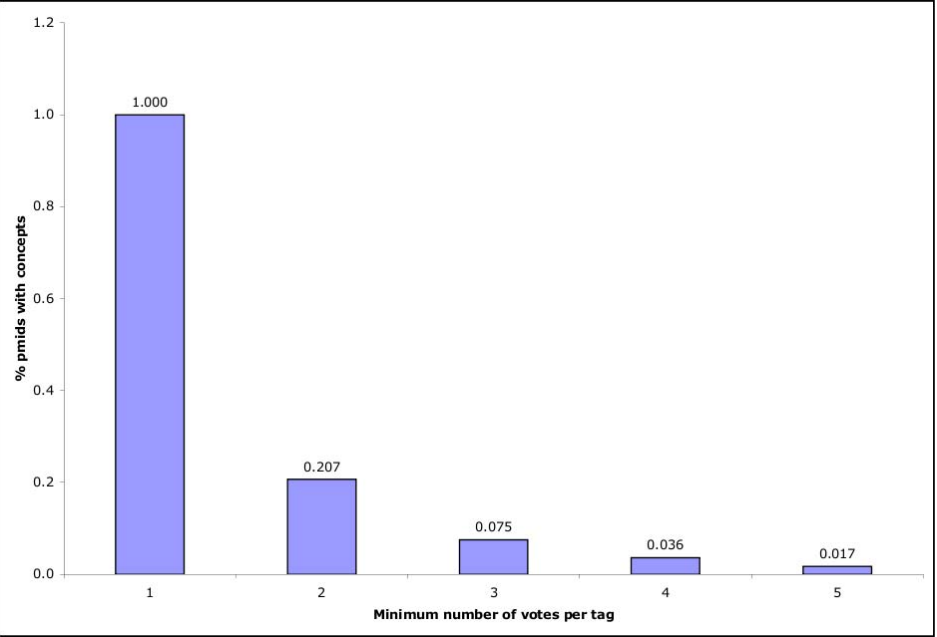
**Precision increase and coverage decrease with voting in CiteULike**. The X axis indicates the minimum number of times a given UMLS Concept would need to be associated with a PubMed citation (through the assignment of a tag by a CiteULike user that could be linked to the Concept) to be considered. If no concepts can be identified for a particular document at each threshold, the document is removed from consideration. The Y axis shows the fraction of PubMed citations associated with UMLS Concepts at each threshold. Only Concepts are plotted as each Concept is linked to a Semantic Type and a Semantic Group hence the other plots would be redundant.

To test this assumption in this context, we investigated the effect of voting on the precision of the concepts linked to tags in the CiteULike system with respect to MeSH indexing. (Once again Connotea was very similar to CiteULike.) Figure 12 illustrates the improvements in precision gained with the requirement of a minimum of 1 through 5 'votes' for each Concept, Semantic Type, or Semantic Group assignment. As the minimum number of required votes increases from 1 to 4, precision increases in each category. At a minimum of 5 votes, the precision of semantic types and semantic groups continues to increase, but the precision of individual concepts drops slightly from 0.335 to 0.332. We did not measure beyond five votes because, as the minimum number of required votes per tag increases, the number of documents with any tags drops precipitously. For documents with no tags, no measurements of agreement can be made. Figure 13 illustrates the decrease in citation coverage associated with increasing minimum numbers of votes per tag assignment. Requiring just two votes per tag eliminates nearly 80% of the citations in the CiteULike collection. By 5 votes, only 1.7% of the citations in the dataset can be considered. This reiterates the phenomenon illustrated in Figures 6, 7, 8 and 9 - at present, most PubMed citations within academic social tagging systems are only tagged by one or a few people.

### An anecdotal example where many tags are present

Though the bulk of the socially generated metadata investigated above is sparse - with most items receiving just a few tags from a few people - it is illuminating to investigate the properties of this kind of metadata when larger amounts are available both because it makes it easier to visualize the complex nature of the data and because it suggests potential future applications. Aside from enabling voting processes that may increase confidence in certain tag assignments, increasing numbers of tags also provide additional views on documents that may be used in many other ways. Here, we show a demonstrative, though anecdotal example where several different users tagged a particular document and use it to show some important aspects of socially generated metadata - particularly in contrast to other forms of indexing.

**Figure 14:**
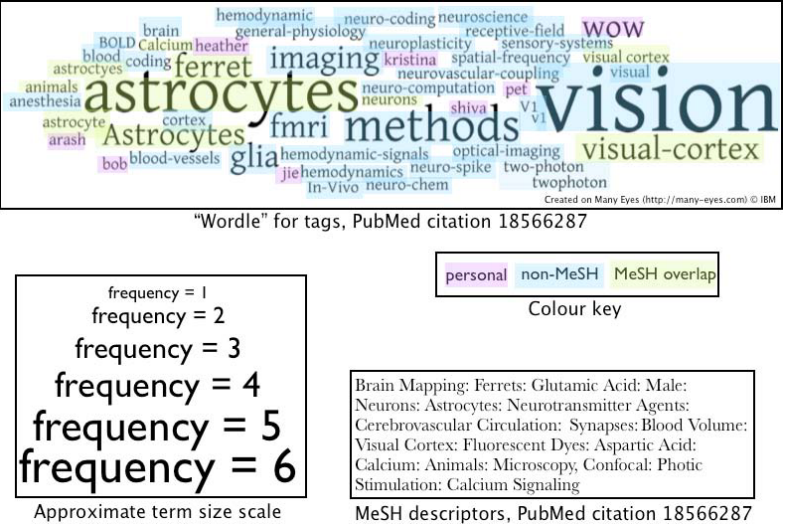
**Tags for a popular PubMed citation from Connote and CiteULike**. The tag cloud or "Wordle" at the top of the figure shows the tags from both CiteULike and Connotea for the *Science *article "Tuned responses of astrocytes and their influence on hemodynamic signals in the visual cortex" (PubMed id 18566287). As the frequency scale at the bottom left indicates, the tags are sized based on the number of times they were used to describe the article. As the colour key at middle-right shows, the tags are divided into three, manually assigned categories: 'personal', 'non-MeSH', and 'MeSH overlap'. Personal tags are those, like 'kristina', that do not appear topical, 'non-MeSH' tags appear topical but do not match directly with any of the MeSH descriptors for the article (listed on the bottom-left), and the 'MeSH overlap' tags have matches within the MeSH descriptors assigned to the article.

Figure 14 illustrates the tags generated by users of Connotea and CiteULike to describe an article that appeared in *Science *in June of 2008 [28]. In the figure, the different tags are sized based on their frequency and divided into three differently coloured classes: 'personal', 'non-MeSH', and 'MeSH Overlap'. The MeSH descriptors for the document are also provided. The figure shows a number of important characteristics of social tagging given current implementations. There are personal tags like 'kristina' and 'bob' but the majority of the tags are topical - like 'neuro-computation'. There are spelling errors and simple phrasing differences in the tags; for example, 'astroctyes, 'astrocytes', 'Astrocytes', and 'astrocyte' are all present (highlighting some of the difficulties in mapping tag strings to concepts). The more frequently used tags ('astrocytes', 'vision', 'methods') are all of some relevance to the article (entitled "Tuned responses of astrocytes and their influence on hemodynamic signals in the visual cortex"). There is some overlap with MeSH indexing but many of the tags - such as 'receptive-field', 'V1', and 'neurovascular-coupling' - that do not match directly with MeSH descriptors also appear to be relevant to the article.

In some cases, the tags added by the users of the social tagging systems are more precise than the terms used by the MeSH indexers. For example, the main experimental method used in the article was two-photon microscopy - a tag used by two different social taggers (with the strings 'two-photon' and 'twophoton'). The MeSH term used to describe the method in the manuscript is 'Microscopy, Confocal'.

Within the MeSH hierarchy, two-photon microscopy is most precisely described by the MeSH heading 'Microscopy, Fluorescence, Multiphoton' which is narrower than 'Microscopy, Fluorescence' and not directly linked to 'Microscopy, Confocal'; hence it appears that the social taggers exposed a minor error in the MeSH annotation. In other cases, the social taggers chose more general categories - for example, 'hemodynamics' in place of the more specific 'blood volume'.

The tags in Figure 14 show two important aspects of socially generated metadata: diversity and emergent consensus formation. As increasing numbers of tags are generated for a particular item, some tags are used repeatedly and these tend to be topically relevant; for this article, we see 'astrocytes' and 'vision' emerging as dominant descriptors. In addition to this emergent consensus formation (which might be encouraged through interface design choices) other tags representing diverse user backgrounds and objectives also arise such as 'hemodynamic'. 'neuroplasticity', 'two-photon', and 'WOW'. In considering applications of such metadata, both phenomenon have important consequences. Precision of search might be enhanced by focusing query algorithms on high-consensus tag assignments or by enabling Boolean combinations of many different tags. Recall may be increased by incorporating the tags with lower levels of consensus.

While we assert that this anecdote is demonstrative, a sample of one is obviously not authoritative. It is offered simply to expose common traits observed in the data where many tags have been posted for a particular resource.

## Discussion

The continuous increase in the volume of data present in the life sciences, illustrated clearly in Figure 2 by the growth of PubMed, renders processes that produce value-enhancing metadata increasingly important. It has been suggested by a number of sources that social tagging services might generate useful metadata, functioning as an effective intermediate between typically inexpensive, but low precision automated methods and expensive professional indexing involving controlled vocabularies [15,21,22,29]. Evidence in favour of this claim comes from reported improvements in the relevance of Web search results gained by integrating information from social tagging data into the retrieval process [17]. Where a substantial density of socially generated tags is present, we demonstrated that it is possible to achieve both deep resource descriptions (Figure 14) and improvements in annotation precision via aggregation (Figure 12). Unfortunately, the results presented here also suggest that much of this potential is as yet unavailable in the context of the life sciences because the coverage of the domain is still very narrow and the number of tags used to describe most of the documents is generally very low.

If metadata from social tagging services is to be useful in support of applications that are similar in purpose and implementation to those currently in operation, more documents need to be tagged and more tags need to be assigned per document. These objectives can be approached by both expanding the number of users of these systems and adjusting the interfaces that they interact with. Looking forward, the increasing volume of contributors to social tagging services should help to increase resource coverage and, to some extent, tag density, yet both the rich-get-richer nature of citation and the limited actual size of the various sub-communities of science will likely continue to result in skewed numbers of posts per resource. To make effective use of the annotations produced by social tagging applications, the metadata generated by individual users needs to be improved in terms of density and relevance because, in most cases, the number of people to tag any particular item will be extremely low. Identifying design patterns that encourage collectively useful tagging behaviour is thus a critical area for future investigations. It has been shown that careful interface and interaction design can be used to guide individual users towards tagging behaviours that produce more useful metadata at the collective level [30,31]. Future research will help to provide a better understanding of this process, illuminating methods for guiding user contributions in particular directions, *e.g*. towards the use of larger numbers of more topical tags, without reducing the individual benefits of using these systems that seem to provide the primary incentive for participation [32]. One such experiment in interaction design would be to inform the users of these systems that the annotations they create for themselves are to be used in the creation of applications that operate on a collective level and thus benefit the community as a whole. By making the desire to create such applications known and by explaining the attributes of the annotations required to make these applications effective, it is possible that some individuals might act intentionally to improve the collective product. Such an experiment would help to shed light on the question of why there are such differences between the tagging behaviours of typical users and the annotations produced in professional contexts. Perhaps an increased overlap in purpose would result in increased overlap in product.

Aside from such overt requests, changes to the interfaces used to author annotations within social tagging systems might also have substantial effects. One key area of development in terms of tagging interface design is the incorporation of controlled vocabularies into the process. Emerging systems in this domain let users tag with controlled terms [33,34] and automatically extract relevant keywords from text associated with the documents to suggest as potential tag candidates [35]. By providing the well-known benefits of vocabulary control - including effective recognition and utilization of relationships such as synonymy and hyponymy - and by gently pressing users towards more convergent vocabulary choices and fewer simple spelling errors, such systems seem likely to produce metadata that would improve substantially on that analyzed here. In preliminary investigations of such 'semantic social tagging' applications - including Faviki [34], the Entity Describer [36,37], and ZigTag [33] - the degrees of inter-tagger agreement do appear higher than for the free-text interfaces however the number of tags per document remains about the same (data not shown). Systems that aid the user in selecting tags - for example, by mining them from relevant text - may aid in the expansion of the number of tags added per document.

In addition to recruiting more users and producing interfaces that guide them towards more individually and collectively useful tagging behaviours, additional work is needed to better understand other aspects of the metadata from social tagging systems that are both important and completely distinct from previous forms of indexing. For example, one of the fundamental differences between socially generated and institutionally generated indexes is the availability of authorship information in the social data [38]. It is generally not possible to identify the person responsible for creating the MeSH indexing for a particular PubMed citation, but it is usually possible to identify the creator of a public post in a social tagging system. This opens up whole new opportunities for finding information online whose consequences are little understood. For example, it is now possible for users to search based on other users *e.g*. searching for items in Connotea that have been tagged by 'mwilkinson' [39] or 'bgood' [40]. In addition to this simple yet novel pattern of information interaction, research is being conducted into ways to incorporate user-related data into keyword-based retrieval algorithms [41].

## Conclusion

Academic social tagging systems provide scientists with fundamentally new contexts for collaboratively describing, finding, and integrating scientific information. In contrast to earlier forms of personal information management, the public nature and open APIs characteristic of social tagging services make the records of these important scientific activities accessible to the community. These new public metadata repositories provide a novel resource for system developers who wish to improve the way scientists interact with information.

Based on the results presented above, it is clear that the information accumulating in the metadata repositories generated through social tagging offers substantial differences from other kinds of metadata. In particular, both the number of documents described by these systems and the density of tags associated with each document remain generally very low and very unequally distributed across both the user and the document space. While expanding numbers of user-contributors and improving user interfaces will likely help to encourage the formation of greater numbers of tagged documents and more useful tags, the unbalanced distribution of scientific attention will almost certainly result in the continuation of the skewed numbers of taggers (and thus tags) per document displayed in Figures 6, 7, 8 and 9.

At a broad level, the key implication of these results from the standpoint of bioinformatics system design is that - despite surface similarities - these new metadata resources can not be used in the same manner as metadata assembled in other ways. Rather, new processes that make use of the additional social context made accessible through these systems need to be explored. In the long run, it may turn out that the primary benefit of social tagging data might not be found in the relationships between tags and documents as explored here but instead in the information linking documents and tags to users and users to each other.

## Methods

### Data Acquisition

The Connotea data was gathered using the Connotea Web API [42] and a client-side Java library for interacting with it [43]. All tagging events accessible via the API prior to November 10, 2008 were retrieved and, with the exception of a small number lost due to XML parsing errors, stored in a local MySQL database for analysis.

The CiteULike data was downloaded on November 9, 2008 from the daily database export provided online [44]. Once again, the data was parsed and loaded into a local MySQL database for processing.

Once the Connotea and CiteULike data was gathered, the associated PubMed identifiers from both datasets were used to retrieve the PubMed records using a Java client written for the United States National Centre for Biotechnology's Entrez Programming Utilities [45]. This client retrieved the metadata, including MeSH term assignments, for each identifier and stored it in the local database.

### Resource coverage

The coverage of PubMed by Connotea and CiteULike was estimated through inspection of the number of unique identifiers supplied for each posted citation in the downloaded data. Only citations that were linked by the tagging systems to PubMed identifiers were counted.

### Tag density

The data generated for the tag density tables and figures was assembled from the local database using Java programs. The figures were generated using R [46].

### Calculation of Positive Specific Agreement (PSA)

In situations where there is no defined number of negative cases, as is generally the case for the assignment of descriptive tags to documents, PSA has been shown to be an effective measure of inter-annotator agreement [24]. PSA can be calculated for any pair of overlapping sets. Here it is used to compare the degree of overlaps between sets of terms, concepts, semantic types, and semantic groups. If one set is considered to be the standard against which the other set is being measured, then PSA is equivalent to the F-statistic (the harmonic mean of precision and recall) commonly used in the machine learning and information retrieval literature. For two sets S1 and S2, consider the set *a *as the members of the intersection of A and B, *b *as the members of S1 outside of the intersection and *c *as the members of S2 outside of the intersection.



**Equation 1**: Positive Specific Agreement for the members of sets S1, S2 whose intersection is a and where b = S1 excluding a and c = S2 excluding a. For more information, see [24].

To provide an estimation for quality of tag assignments in academic social tagging systems, we measure the levels of agreement between the sets of tags assigned to the same resource by multiple users as follows:

- For resources associated with more than one tagging event

◦ For pairs of users to tag the resource

▪ measure and record the positive specific agreement (PSA) between the tags assigned to the resource between the pair

- Summarize by average PSA for each distinct (user-pair, resource) combination

### String standardization for tag comparisons

As PSA is a metric designed for comparing sets, to use it, it is necessary to define a rigid equivalence function to define the members of the sets. For comparisons between concepts, types, and groups from the UMLS, unique identifiers for each item are used; however, for comparisons between tags, only the strings representing the tag are available. For the results presented at the level of standardized strings, operations were applied to the tags prior to the comparisons as follows:

1. All non-word characters (for example, commas, semi-colons, underscores and hyphens) were mapped to spaces using a regular expression. So the term "automatic-ontology_evaluation" would become "automatic ontology evaluation".

2. CamelCase [46] compound words were mapped to space separated words - "camelCase" becomes "camel case".

3. All words were made all lower case ("case-folded").

4. Any redundant terms were removed such that, after operations 1-3, each term in a set composed a string of characters that was unique within that set.

5. Porter stemming was applied to all terms and sub-terms [47].

6. All sub-terms were sorted alphabetically.

### Mapping tags and descriptors to UMLS concepts

For MeSH terms, associated UMLS concepts were identified within the information provided in the 2008 version of the MeSH XML file provided by the NLM [48]. In a few cases, concepts were missing from this file in which case they were retrieved using a Java client written to make use of the Web services made available as part of the UMLS Knowledge Source Server (UMLSKS) [49].

For the tags, the UMLSKS client program was designed to identify matching concepts with high precision. For each tag, the UMLSKS web service method *findCUIByExact *was used to identify concepts from any of the source vocabularies represented in the metathesaurus where at least one of the names assigned to that concept matched the tag directly [50]. To further increase precision, only concepts whose primary name (rather than one of the several possible alternate names) matched the tag were included.

To assess the performance of this concept identification protocol, we tested it on its ability to rediscover the concepts associated with MeSH descriptors using the text of the preferred label for the descriptor (acting as a tag) as the input to the system. The concepts already associated with each MeSH descriptor in the MeSH XML file provided by the NLM were used as true positive concept calls for comparison. On a test of 500 MeSH descriptors, the concept calling protocol used to generate the data presented above produced a precision of 0.97 and a recall of 0.91. Without the requirement that the primary concept name match the query string, precision decreases to 0.82 while the recall increases to 1.0 for the same query set. The reduction in the precision is due to false positives such as 'Meningeal disorder' being identified for the query term 'Meninges'. Once a unique concept identifier was identified, the Java client was used to extract its semantic type and semantic group and store this information in our local database.

## Authors' contributions

BMG conceived of the study, implemented all analyses and required software, and drafted the manuscript. JTT and MDW contributed conceptually to the study and helped to draft the manuscript. All authors read and approved the final manuscript.

## References

[B1] Use of MeSH in Indexing. http://www.nlm.nih.gov/mesh/intro_indexing2007.html.

[B2] Bachrach C, Charen T (1978). Selection of MEDLINE contents, the development of its thesaurus, and the indexing process. Medical Informatics.

[B3] Hubbard T, Aken B, Beal K, Ballester B, Caccamo M, Chen Y, Clarke L, Coates G, Cunningham F, Cutts T (2007). Ensembl 2007. Nucleic acids research.

[B4] Ruch P (2006). Automatic assignment of biomedical categories: toward a generic approach. Bioinformatics.

[B5] Aronson A, Bodenreider O, Chang H, Humphrey S, Mork J, Nelson S, Rindflesch T, Wilbur W (2000). The NLM Indexing Initiative. Proceedings of the AMIA Annual Symposium; Los Angeles.

[B6] Kim W, Aronson A, Wilbur W, Susan Bakken (2001). Automatic MeSH term assignment and quality assessment. Proceedings of the AMIA Annual Symposium; Washington DC.

[B7] Gattiker A, Michoud K, Rivoire C, Auchincloss A, Coudert E, Lima T, Kersey P, Pagni M, Sigrist C, Lachaize C (2003). Automated annotation of microbial proteomes in SWISS-PROT. Computational biology and chemistry.

[B8] Kasukawa T, Furuno M, Nikaido I, Bono H, Hume D, Bult C, Hill D, Baldarelli R, Gough J, Kanapin A (2003). Development and Evaluation of an Automated Annotation Pipeline and cDNA Annotation System. Genome Research.

[B9] Lund B, Hammond T, Flack M, Hannay T (2005). Social Bookmarking Tools (II): A Case Study - Connotea. D-Lib Magazine.

[B10] CiteULike: A free online service to organize your academic papers. http://www.citeulike.org/.

[B11] Del.icio.us. http://del.icio.us/.

[B12] Flickr. http://www.flickr.com/.

[B13] 43Things. http://www.43things.com/.

[B14] Golder SA, Huberman BA (2006). Usage patterns of collaborative tagging systems. Journal of Information Science.

[B15] Hammond T, Hannay T, Lund B, Scott J (2005). Social Bookmarking Tools (I): A General Review. D-Lib Magazine.

[B16] Hotho A, Jäschke R, Schmitz C, Stumme G, de Moor A, Polovina S, Delugach H (2006). BibSonomy: A Social Bookmark and Publication Sharing System. Proceedings of First Conceptual Structures Tool Interoperability Workshop at the 14th International Conference on Conceptual Structures: July 16, 2006; Aalborg.

[B17] Morrison PJ (2008). Tagging and searching: Search retrieval effectiveness of folksonomies on the World Wide Web. Information Processing and Management.

[B18] Worio Search. http://www.worio.com/.

[B19] Key MEDLINE Indicators. http://www.nlm.nih.gov/bsd/bsd_key.html.

[B20] Wheeler DL, Barrett T, Benson DA, Bryant SH, Canese K, Chetvernin V, Church DM, Dicuccio M, Edgar R, Federhen S (2008). Database resources of the National Center for Biotechnology Information. Nucleic acids research.

[B21] Kipp MEI, Arsenault C, Dalkir K (2007). Tagging practices on research oriented social bookmarking sites. Proceedings of the 35th Annual Conference of the Canadian Association for Information Science: May 10 - 12, 2007 Montreal.

[B22] Kipp MEI, Tennis JT (2007). Tagging for health information organization and retrieval. Proceedings of the North American Symposium on Knowledge Organization: June 14-15, 2007 Toronto.

[B23] Kipp MEI (2006). @toread and Cool: Tagging for Time, Task and Emotion. Proceedings of the 7th Information Architecture Summit March 23-27, 2006 Vancouver.

[B24] Hripcsak G, Rothschild AS (2005). Agreement, the F-Measure, and Reliability in Information Retrieval. Journal of the American Medical Informatics Association: JAMIA.

[B25] Bodenreider O (2004). The Unified Medical Language System (UMLS): integrating biomedical terminology. Nucl Acids Res.

[B26] McCray AT (2003). An upper-level ontology for the biomedical domain. Comp Funct Genomics.

[B27] McCray AT, Burgun A, Bodenreider O (2001). Aggregating UMLS semantic types for reducing conceptual complexity. Stud Health Technol Inform.

[B28] Schummers J, Yu H, Sur M (2008). Tuned responses of astrocytes and their influence on hemodynamic signals in the visual cortex. Science.

[B29] Tagging, Folksonomy & Co - Renaissance of Manual Indexing?. http://arxiv.org/abs/cs/0701072v1.

[B30] Sen S, Lam SK, Rashid AM, Cosley D, Frankowski D, Osterhouse J, Harper FM, Riedl J (2006). Tagging, communities, vocabulary, evolution. Proceedings of the 20th anniversary conference on Computer Supported Cooperative Work November 4-8, 2006 Banff.

[B31] Drenner S, Sen S, Terveen L (2008). Crafting the initial user experience to achieve community goals. Proceedings of the ACM Conference On Recommender Systems October 23-25, 2008 Lausanne.

[B32] Bokardo: The Del.icio.us Lesson. http://bokardo.com/archives/the-delicious-lesson/.

[B33] Zigtag. http://www.zigtag.com.

[B34] Faviki - Social bookmarking tool using smart semantic Wikipedia (DBpedia) tags. http://www.faviki.com.

[B35] Twine - Organize, Share, Discover Information Around Your Interests. http://www.twine.com.

[B36] The Entity Describer. http://www.entitydescriber.org.

[B37] Good BM, Kawas EA, Wilkinson MD (2007). Bridging the gap between social tagging and semantic annotation: E.D. the Entity Describer. Nature Precedings.

[B38] Tennis JT (2006). Social Tagging and the Next Steps for Indexing. Proceedings of the 17th ASIS&T SIG/CR Classification Research Workshop November 3 2006 Austin.

[B39] mwilkinson's bookmarks. http://www.connotea.org/user/mwilkinson.

[B40] bgood's bookmarks. http://www.connotea.org/user/bgood.

[B41] Abbasi R, Staab S (2008). Introducing Triple Play for Improved Resource Retrieval in Collaborative Tagging Systems. Proceedings of the ECIR Workshop on Exploiting Semantic Annotations in Information Retrieval(ESAIR'08) March 30, 2008 Barcelona.

[B42] Connotea Web API. http://www.connotea.org/wiki/WebAPI.

[B43] Java Client for Connotea API. http://code.google.com/p/connotea-java/.

[B44] Citeulike database export. http://www.citeulike.org/faq/data.adp.

[B45] Sayers EW, Barrett T, Benson DA, Bryant SH, Canese K, Chetvernin V, Church DM, Dicuccio M, Edgar R, Federhen S (2008). Database resources of the National Center for Biotechnology Information. Nucleic Acids Res.

[B46] R development Core Team R: A language and environment for statistical computing. http://www.R-project.org.

[B47] Porter MF (1980). An algorithm for suffix stripping. Program.

[B48] Medical Subject Headings - Files Available toDownload. http://www.nlm.nih.gov/mesh/filelist.html.

[B49] UMLS Knowledge Source Server. http://umlsks.nlm.nih.gov/.

[B50] UMLSKS documentation. http://umlsks.nlm.nih.gov/DocPortlet/html/dGuide/webservices/metaops/find/find1.html.

